# Use of Relational Agents to Improve Family Communication in Type 1 Diabetes: Methods

**DOI:** 10.2196/resprot.5817

**Published:** 2016-07-28

**Authors:** Debbe Thompson, Karen W Cullen, Maria J Redondo, Barbara Anderson

**Affiliations:** ^1^ USDA/ARS Children's Nutrition Research Center Department of Pediatrics Baylor College of Medicine Houston, TX United States; ^2^ Texas Children's Hospital Pediatric Endocrinology and Metabolism Baylor College of Medicine Houston, TX United States; ^3^ Psychology Department of Pediatrics Baylor College of Medicine Houston, TX United States

**Keywords:** adolescents, family communication, pre-adolescents, relational agent, type 1 diabetes

## Abstract

**Background:**

Physiological and environmental risk factors interact to undermine blood glucose control during early adolescence. This has been documented to be associated with family conflict and poor adherence to diabetes management tasks. Family Teamwork is an efficacious program demonstrated to enhance family communication and reduce conflict during this vulnerable period. It was designed to be delivered to families in-person, which limited reach and potential impact.

**Objective:**

The purpose of this paper is to present the protocol for adapting Family Teamwork for Web-based delivery.

**Methods:**

Formative research with health care providers, parents, and adolescents will help modify Family Teamwork for Web-based delivery by a relational agent (ie, a computerized character with human-like features and actions). Sessions will be interactive, requiring both parent and adolescent participation, with the relational agent serving as a health coach. After programming, usability testing will be conducted to help ensure the program is easy to use. Video and instructional materials will be developed to facilitate use, and a small pilot study will be conducted to assess feasibility. Families will provide written informed consent prior to participation in any phase of the study. The Institutional Review Board at Baylor College of Medicine reviewed and approved the protocol (H-37245).

**Results:**

Formative research is underway. No results are available at this time.

**Conclusions:**

This research has the potential to make an important contribution to diabetes management by using technology to enhance the reach of an efficacious program.

## Introduction

The incidence of type 1 diabetes (T1D) is increasing worldwide [1], and T1D is the second most prevalent chronic illness among US children, after asthma [2]. Despite the recent introduction of new types of insulin, insulin delivery systems, and innovative blood glucose (BG) monitoring technologies to improve T1D self-management and BG control, non-adherence to a diabetes management regimen remains common, especially in young adolescents with T1D [3]. Unfortunately, physiological and environmental risk factors interact to undermine BG control during pre- and early adolescence. While the physiologic insulin resistance that occurs normally during pubertal development and resulting deterioration of BG control have been well-established [4], only recently have investigators documented the significant role of the family in diabetes adherence and BG control during this period [5]. Recent longitudinal studies [6-8] have demonstrated that poor adherence and BG control during adolescence, as well as family problems, often persist into early adulthood, amplifying the risk of long-term microvascular, macrovascular, and psychological complications. Therefore, it is increasingly clear that the pre- and early-adolescence periods are particularly critical. Intervening during this period is essential for improving both adherence and diabetes-specific family interactions, which will establish a trajectory of strong, stable self-management behavior and more optimal BG during adolescence, thus lowering the risk for long-term complications [9,10].

Family Teamwork (FT) is a clinic-based face-to-face intervention for pre- and early-adolescent youth with T1D and their parents. It targets potentially modifiable factors documented to impact glycemic control and adherence to BG monitoring, such as parent-youth conflict and communications around BG monitoring. FT was designed to increase positive parent involvement in, and reduce family conflict around, T1D management in young adolescents with T1D. Its goal was to improve adherence and BG control as reflected by hemoglobin A1c (HbA1c) [11]. The 8-session program was delivered to 10-14 year-olds and a parent during routine clinic visits by a trained research assistant. Two randomized controlled trials demonstrated its efficacy (ie, significant improvement in BG monitoring adherence and HbA1c in the FT group compared with the standard care group [11,12], as well as increases in self-reported quality of life [13]). Parents in the FT group maintained or increased involvement in diabetes management tasks, especially BG monitoring, with no increase in diabetes-specific family conflict [14]. Youth in the intervention arm improved BG monitoring adherence [11] and self-reported quality of life [13]. Furthermore, participants who received the intervention had a decrease in HbA1c from 8.4% ±1.3% to 8.2% ±1.1% compared with the deterioration from 8.3% ±1.0% to 8.7% ±1.5% (*P*<.05) observed in the control group, as expected during early adolescence [12].

Even though FT was proven to be efficacious, its reach was severely limited by the need for families to travel to a particular location to participate in the intervention and the costs associated with delivery by a trained research assistant. Since there is an urgent need to broadly disseminate effective interventions for the high-risk group of early adolescent youth with T1D [11], a method to deliver FT in a more convenient, lower-cost format is needed. Internet use is prevalent in today’s world [15]. Therefore, adapting FT for delivery via a Web-based format, led by a relational agent (an animated computer character with human-like features and behaviors) may offer a solution.

Research has demonstrated the feasibility and acceptability of relational agents. For example, relational agents have been utilized in a variety of adult populations and with a wide array of health behaviors (eg, a virtual nurse providing discharge instructions to low health literate patients [16] and patients with depressive symptoms [17]; an exercise advisor for college students [18,19], adults [20], and low health literate older adults [16,18,21]; a health advisor promoting medication adherence to adults with schizophrenia [18,22]; a virtual coach promoting adherence to physical activity in overweight adults [23]; and a virtual agent promoting fruit and vegetable consumption to healthy adults [20]). They are also being developed for use in group settings and for multiple behaviors. Because research shows promising evidence that relational agents can establish a therapeutic relationship with patients and that they are well accepted by a variety of patient populations [18,19], this approach has potential as a method for overcoming limitations commonly associated with face-to-face behavioral interventions, such as limited reach, scheduling constraints, and variable fidelity [24,25]). Thus, incorporating relational agents into programs traditionally delivered in-person could overcome these limitations and provide a low-cost, easy-to-disseminate method for reaching families in need.

This research will convert FT to a Web-based delivery format guided by a relational agent (ie, Family Teamwork Online [FTO]) and assess the feasibility of this approach. This research addresses an important gap in the field and has the potential to enhance the reach and potential impact of a proven, efficacious intervention developed for an at-risk group. The purpose of this paper is to describe the protocol for adapting FT to a Web-based format guided by a relational agent.

## Methods

### Overview

This research will be conducted in two phases: development and pilot. The purpose of the development phase is to conduct formative research with parents and adolescents with T1D and their providers in order to adapt the program to a Web-based format. The pilot phase will assess feasibility of this approach. Each phase is described below. Ethical approval was provided by the Institutional Review Board at Baylor College of Medicine (H-37245). Because the purpose of this trial is to establish the feasibility of this approach versus a randomized control trial to determine efficacy or effectiveness, the trial has not been registered with a trial registry accredited by the World Health Organization.

### Theoretical Framework

**Figure 1:**
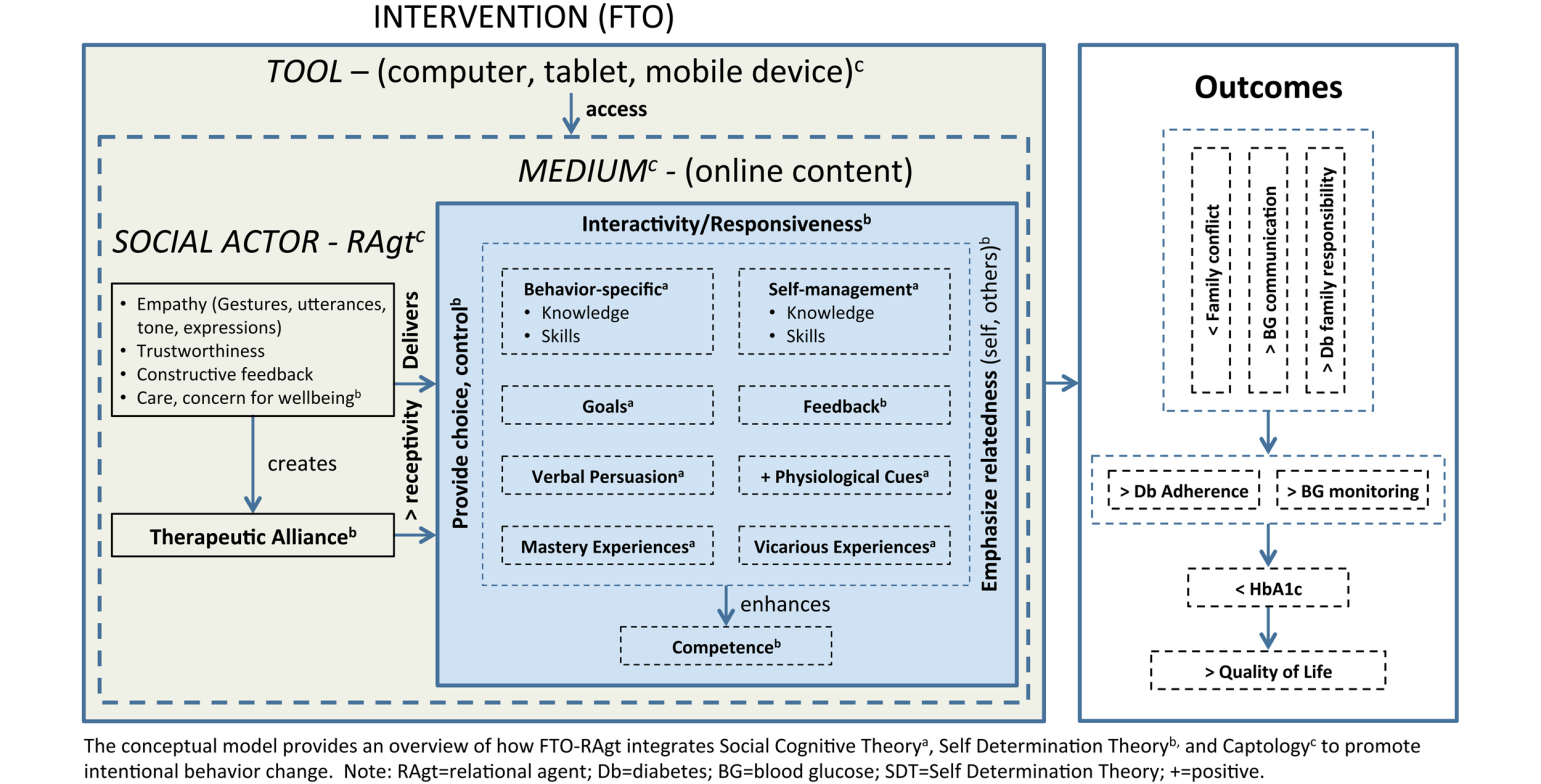
Conceptual Model of how FTO is designed to influence outcomes.

The content and structure of the original FT was grounded in social cognitive theory (SCT) [26]. The adaptation of FT to FTO will be guided by Computers As Persuasive Technologies (CAPTOLOGY) [27] and self-determination theory (SDT) [28]. CAPTOLOGY provides a framework for understanding how computers can be used as a persuasive mechanism to intentionally change attitudes and behaviors. For example, computers can personalize the encounter (eg, greeting family members by name), provide an interactive versus didactic session, simulate experiences (eg, provide opportunities for the parent/adolescent dyad to practice skills taught in the session), and receive tailored feedback based on responses, problems, or issues brought up in the session [27]. The framework posits that this is achieved through the “functional triad,” which is a unique combination of the tool (eg, access device, such as a computer or tablet), medium (eg, delivery mode, such as the Internet), and social actor (eg, relationship builder, such as the relational agent) [27]. SDT [28] contends that three basic needs drive behavior: competence (ie, knowledge, skills, ability to successfully perform a behavior), autonomy (ie, choice, control), and relatedness (ie, connection to important others). A high level of need satisfaction promotes internalization and integration of the behavior into one’s sense of self (ie, “I am a person who routinely monitors my BG,” “I am a person who tries to understand my parent’s perspective when we disagree over my diabetes”). Internalization and integration of a behavior with one’s sense of self increases internally driven motivation to perform the behavior. This, in turn, increases the likelihood that the behavior will be performed and maintained over time [28]. The relational agent will be constructed to emphasize need fulfillment. For example, it will enhance effective communication among parents and adolescents around T1D self-management behaviors by presenting skills, encouraging practice (ie, competence), and emphasizing personal choice regarding how they interpret comments and respond to each other (ie, autonomy). Improved communication will provide insight into what the other person’s motivations may be when they react in a certain way, and it will help establish a bond of trust and rapport with the relational agent (ie, relatedness). Figure 1 shows the conceptual model guiding the adaptation.

### Study Sample

Health care providers at a diabetes care center in a large tertiary care children’s hospital in the southwestern United States are eligible if they are employed full or part time by the facility. They will be invited by email to participate in the first phase of the study.

Current patients and their primary diabetes caregivers attending a large diabetes care center in Texas are eligible to participate in this study: 10-14 year olds with T1D as defined by the American Diabetes Association criteria [29], disease duration at least 1 but not over 5 years, fluent in English with access to high-speed Internet, and a parent willing to participate in the study are eligible to participate. Adolescents are ineligible if the average HbA1c over the past year is ≥12% (due to a greater likelihood of having psychiatric conditions [30]) or ˂7% (excellent glycemic control), unable to attend regular clinic visits, or have a physical/mental disease or condition that may conflict with study protocol and limit ability to complete data collection activities or participate in the intervention.

Eligible parents must be the primary caregiver of a child with T1D enrolled in the study, be willing to participate in study activities, be fluent in English, have access to high-speed Internet, and not be planning to leave the geographic area.

### Recruitment

To identify families, a research coordinator experienced in working with families with diabetes will screen the clinic appointment schedule to identify families who meet the eligibility criteria. Eligible families will be invited by letter to participate. Within a week of sending the letter, study staff will contact the families to answer questions, ascertain interest, and screen for eligibility. If families are interested and eligible, written informed consent and child assent will be obtained.

### Development Phase

The purpose of this phase is to conduct formative research to adapt FT for Web-based delivery by a relational agent. It consists of in-person interviews with health care providers, Web-based surveys and telephone interviews with parents and adolescents, in-person usability testing with parents and adolescents, and development of a brief instructional video and supporting materials to facilitate intervention completion.

#### Sample Sizes

A purposive sampling approach will be used to identify sample sizes for the formative research [31]. We selected this approach expecting that it would provide key insights from stakeholders (health care providers, families who have a child with T1D) that could be used to update the content and develop a program sensitive to the needs of families enrolled in the study. In this sampling approach, sample size is driven by the number of participants needed to address key research questions. Therefore, formative research will involve 10 health care providers and up to 24 parent/adolescent dyads. Usability testing will be conducted with a different sample of up to 12 parent/adolescent dyads. If analysis does not yield adequate information with which to address the research questions, additional data will be collected until this point is attained.

#### Health Care Providers

Health care providers will participate in a scripted, semistructured interview to identify their general thoughts about FTO, diabetes management concerns, and issues often seen in clinic related to family conflict. Interviews will be digitally recorded and transcribed verbatim. Data will be coded and analyzed using thematic analysis [32]. A priori codes will provide the initial coding framework; they will be augmented with additional codes that emerge during analyses. Codes will be examined to identify themes and patterns. Discrepancies will be discussed and resolved.

#### Parent/Adolescent Dyads

Formative research with families will include up to two Web-based surveys, each followed by a telephone interview to clarify, expand, and understand survey responses. Parents and adolescents will participate in this phase separately. They will be asked to provide feedback on the relational agent (eg, looks, clothing, skin tone, hair style, name, facial expressions), issues their family commonly faces surrounding diabetes management, usual reactions, and suggested session topics. This information will be used to adapt FT for Web-based delivery (FTO) and to develop the relational agent. Sample questions will include, regarding relational agent mock-ups, “Which virtual health educator appeals to you the most?” (response options will include Male, Female with curly hair, Female with straight hair); relating to structure, “The sessions will be delivered online through your computer. Parents and children will view the program together. In your opinion, about how long should each session last?” (response options will range from 15 minutes-1 hour); and regarding content, “What is your [parent’s/teen’s] usual reaction to high blood glucose readings?” (response options will include Calmly talks about it, Refuses to talk about it, Gets upset or angry, Gets frustrated, Gets defensive, None apply).

#### Creating Family Teamwork Online

The information presented in each content segment, including content, questions, response options, and feedback will be adapted from the original FT for Web-based delivery by a relational agent based on feedback from health care providers and families. Each session will focus on a specific topic informed by the original FT and the formative research. Sessions will be scripted and will include (1) didactic components where the relational agent conveys content, components where “typical” family scenarios are demonstrated, and (2) interactive components where the relational agent poses a question for the families, parent, and/or adolescent, they select a response, and the relational agent responds. Parents and adolescents will view the sessions together. Session delivery will mimic the original FT delivery by a trained research coordinator as closely as possible. For example, during each session, the relational agent will convey the session content in segments, rather than all at once. Similar to in-person delivery, after presenting content, the relational agent will ask the family a question. The family will have several response options from which to select. Then feedback will be provided. Responses will be made via mouse click. This pattern will continue until the end of the session, which ends in a joint goal-setting task specific to family communication around T1D management. The relational agent’s verbal and nonverbal behaviors will be generated using pre-rendered three-dimensional animated video clips. The video clips will be based on scripts generated for each session. Responses will trigger the next video clip in the sequence. The list of questions is set and will not branch.

FTO will be programmed to be viewed over a high-speed Internet connection, from a desktop, laptop, tablet, or mobile device. It will include high-resolution graphics and vocal tracks, animation, and interactivity. Because it is being programmed to be viewed online, the program will not be device dependent.

#### Usability Testing

After development, FTO usability (ie, ease of use) will be assessed with up to 12 new families to identify technical issues and ease of navigation (ie, do parents/adolescents understand what to do and can they do it without assistance). Following standard usability procedures [33], research staff will observe and keep a log of difficulties as participants (parent/adolescent dyads) work through sessions. On completion, the retrospective think-aloud technique will be used to guide the family through a description of what they did, why, problems they encountered, and how they addressed them as they navigated the program. The research coordinator will take notes of their comments. When the parent/child dyad has finished, using the retrospective probing technique, the research coordinator will ask questions about their thoughts and actions based on the notes taken during the observation and think aloud sessions. Each parent and adolescent will also complete the System Usability Scale [34]; a score of >80.3 will be interpreted to mean that the system has a high level of usability [35].

#### Instruction

A brief video and colorful print information guide will be developed demonstrating how to navigate FTO. These materials will be written at a 5^th^ grade reading level to facilitate comprehension by both parents and adolescents.

### Pilot Phase

#### Sample Size

Feasibility studies are designed to contribute to a well-informed main trial [36-38] and are the first step in intervention development [37-39]. Although the literature does not offer consistent guidance, an appropriate sample size should represent the minimum number of participants needed to adequately assess the feasibility criteria [40]. A sample size of 24 dyads would provide a reasonable evaluation of feasibility; it would also be large enough to examine trends in HbA1c over time.

#### Design

The feasibility study will use a one-group design with three data collection periods: baseline, post 1 (immediately after completion of the online program, ie, approximately 3 months after baseline), and post 2 (approximately 3 months after post 1, ie, approximately 6 months after baseline). Because the primary outcome in a future efficacy study will include HbA1c, the pilot study will encompass 6 months. This will enable an examination of trends in intervention effects on HbA1c over time.

#### Procedure

FTO will be completed online using procedures from other online studies [41,42]. Parents/adolescents will complete the sessions together; they will each be given unique passwords to log on to the program. Both parent and adolescent will need to log on to view a new session. Families will receive email reminders when eligible to log on to the next session. Clinical data collection will occur during the usual clinic visits, online, and as parents/adolescents navigate FTO. Each session will be led by the relational agent who will work with the parent/adolescent dyad during the program. At the end of each session, families will have the option to print their goal and a tip sheet offering suggestions for ways to enhance goal attainment. Families can replay previously viewed sessions unlimited times.

#### Data Collection Procedure and Measures

Several types of data will be collected during this study. Self-report questionnaires will be completed by parents/adolescents separately over a secure, password-protected website at baseline, post 1, and post 2. Trained research staff will extract clinic data needed for the study from the medical record following approved clinic procedures. Program use data will be automatically collected as families navigate FTO. Staff logs will be maintained to assess key process evaluation variables (see Table 1; [17,43-49]).

#### Feasibility Outcomes

FTO will be considered feasible if (1) recruitment goals are met, (2) families complete ≥75% of the sessions (ie, login rate), (3) attrition rate is ≤10%, (4) program satisfaction with FTO is high (average score of ≥16/20), (5) therapeutic alliance with the relational agent is high (average score of 5/7), (6) families express positive attitudes toward the relational agent (average score of 5/7), (7) ≥80% of data are collected at post 1 and post 2, and (8) few technical issues (<10%) with intervention delivery occur.

#### Analysis Plan

##### Feasibility

Analysis for the feasibility study will be mainly descriptive. To enrich understanding of the FTO process, descriptive statistics will be calculated and compared to the target goals. FTO will be considered feasible if target goals are met.

##### Exploratory

Using a within-subject design, linear effect mixed models will examine change in HbA1c and self-report psychosocial measures over time (ie, baseline to post 1, post 2), controlling for potential confounders (eg, gender, race/ethnicity). Separate models will be conducted for psychological and behavioral outcomes. Self-report outcomes will be analyzed separately for parents and adolescents. Although statistical significance is not expected due to the small sample size, changes will be examined to determine if they are in the expected directions. Analyses will be calculated with SAS 9.4 [50].

### Anticipated Results

We anticipate that feasibility criteria will be met and that families in the FTO group will have favorable changes in the expected directions.

**Table 1:** Pilot study measures.

Who	What	Method	Prior	Baseline	Intervention	Post 1	Post 2
Adolescent	Diabetes Self-Management Questionnaire [43]	Self-report		x		x	x
	Peds QL Diabetes Module 3.2 [44]	Self-report		x		x	x
	Revised Diabetes Family Conflict Scale [45]	Self-report		x		x	x
	BG Monitoring Communication Survey [46]	Self-report		x		x	x
	Diabetes Family Responsibility Questionnaire [47]	Self-report		x		x	x
	Program satisfaction [41,48]	Self-report				x	
	Therapeutic Alliance [49]	Self-report				x	
	Attitudes toward Relational Agent [17]	Self-report				x	
	Program reactions	Interview				x	x
	BG meter/insulin pump readings	EHR^a^		x		x	x
	HbA1c	EHR		x		x	x
	Height	EHR		x		x	x
	Weight	EHR		x		x	x
	Treatment regimen	EHR		x		x	x
	Severe hypoglycemia/ketoacidosis	EHR		x		x	x
	Emergency room visits/hospitalizations	EHR		x		x	x
Parent	Revised Diabetes Family Conflict Scale [45]	Self-report		x		x	x
	BG Monitoring Communication Survey [46]	Self-report		x		x	x
	Diabetes Family Responsibility Questionnaire [47]	Self-report		x		x	x
	Demographics	Self-report		x			
	Program satisfaction	Self-report				x	
	Therapeutic Alliance [49]	Self-report				x	
	Attitudes toward Relational Agent [17]	Self-report				x	
	Program reactions	Interview				x	x
Program	Logins	Program			x		
	Responses	Program			x		
	Technical issues	Program			x		
	Recruitment	Staff logs	x				
	Attrition	Staff logs		x	x	x	x
Health care providers	Opinions to help develop FTO	Interview	x				

^a^EHR: electronic health record.

## Discussion

### Principal Considerations

The Diabetes Control and Complications Trial and its findings heightened awareness of the critical importance of maintaining near-normal BG levels to delay and/or prevent T1D complications [51]. Adolescents are particularly affected by poor adherence to the demanding T1D regimen. Family conflict and negative communication around diabetes management, especially around BG monitoring, are barriers to adolescent adherence to their treatment plan [14]. A meta-analysis of pediatric T1D interventions with adherence-promoting components concluded that behavioral interventions focusing “on direct, behavioral processes and neglected emotional, social and family processes are unlikely to have an impact on BG control” (p. 1658) [52]. The most efficacious interventions addressed both [52]. The FT intervention meets these criteria: it targets interactions of the parent and adolescent with T1D and addresses T1D management behaviors (eg, BG monitoring, administering insulin, carbohydrate counting).

Although face-to-face interactions with health care providers have historically been thought of as the most effective method for achieving health behavior change and are considered the “gold standard” [18,53], limited reach [53], time [18], and consistency in intervention delivery can reduce effectiveness [18]. Relational agents may help overcome these limitations. They simulate characteristics of face-to-face interactions with a health care provider, including verbal and nonverbal behaviors that contribute to trust, rapport, and relationship-building. Programs delivered by relational agents are also convenient, accessible, and likely cost effective, particularly when delivered online [18].

Relational agents have been utilized in a variety of populations and health behaviors [16-23] *.* However, to our knowledge, they have not been used to enhance family communication around T1D in adolescence. Because research shows promising evidence that relational agents can establish a therapeutic relationship with patients and that they are accepted by a wide variety of patient populations [18,19], relational agents have the potential to enhance reach and public health impact of efficacious interventions by overcoming limitations associated with face-to-face delivery. Thus, if proven feasible, this research has the potential to ultimately impact how health education programs are delivered to families of adolescents with T1D and other chronic diseases in which effective family communication is essential.

### Limitations

Limitations of this research include conducting the research in one geographic region of the United States, which may limit generalizability. However, this is a pilot study, seeking to establish feasibility and proof of concept, which somewhat overcomes this concern at this stage of intervention development. The sample size is also small; however, once feasibility is established, fully powered efficacy and effectiveness trials can be conducted with larger, more diverse samples. Self-report questionnaires are also used to report psychological information. However, objective measures of adherence will be captured by retrieving BG meter readings and other health outcomes, such as lab values of glycemic control (HbA1c), from the electronic health record. However, whenever possible, gold standard measures will be used in order to obtain the best information possible.

### Conclusions

In conclusion, this research is novel and has the potential to make an important contribution to the scientific literature by expanding the reach and thus the public health impact of programs typically delivered in-person to families that have a child or adolescent with T1D.

## Abbreviations

BG: blood glucose
CAPTOLOGY: Computers as Persuasive Technology
EHR: electronic health record
FT: Family Teamwork
FTO: Family Teamwork Online
HbA1c: hemoglobin A1c
SAS: Statistical Analysis Software
SCT: social cognitive theory
SDT: self-determination theory
T1D: type 1 diabetes
